# Heritage Speakers as Part of the Native Language Continuum

**DOI:** 10.3389/fpsyg.2021.717973

**Published:** 2022-02-09

**Authors:** Heike Wiese, Artemis Alexiadou, Shanley Allen, Oliver Bunk, Natalia Gagarina, Kateryna Iefremenko, Maria Martynova, Tatiana Pashkova, Vicky Rizou, Christoph Schroeder, Anna Shadrova, Luka Szucsich, Rosemarie Tracy, Wintai Tsehaye, Sabine Zerbian, Yulia Zuban

**Affiliations:** ^1^Department of German Language and Linguistics, Humboldt-Universität zu Berlin, Berlin, Germany; ^2^Department of English and American Studies, Humboldt-Universität zu Berlin, Berlin, Germany; ^3^Department of Slavic and Hungarian Studies, Humboldt-Universität zu Berlin, Berlin, Germany; ^4^Leibniz-Zentrum Allgemeine Sprachwissenschaft, Berlin, Germany; ^5^Center for Cognitive Science, Technische Universität Kaiserslautern, Kaiserslautern, Germany; ^6^Department of German, Universität Potsdam, Potsdam, Germany; ^7^Department of English, Universität Mannheim, Mannheim, Germany; ^8^Department of Linguistics, Universität Stuttgart, Stuttgart, Germany

**Keywords:** heritage speakers, registers, participles, word order, bare NPs, boundary tone, referent introduction, relative clause formation

## Abstract

We argue for a perspective on bilingual heritage speakers as native speakers of both their languages and present results from a large-scale, cross-linguistic study that took such a perspective and approached bilinguals and monolinguals on equal grounds. We targeted comparable language use in bilingual and monolingual speakers, crucially covering broader repertoires than just formal language. A main database was the open-access RUEG corpus, which covers comparable informal vs. formal and spoken vs. written productions by adolescent and adult bilinguals with heritage-Greek, -Russian, and -Turkish in Germany and the United States and with heritage-German in the United States, and matching data from monolinguals in Germany, the United States, Greece, Russia, and Turkey. Our main results lie in three areas. (1) We found non-canonical patterns not only in bilingual, but also in monolingual speakers, including patterns that have so far been considered absent from native grammars, in domains of morphology, syntax, intonation, and pragmatics. (2) We found a degree of lexical and morphosyntactic inter-speaker variability in monolinguals that was sometimes higher than that of bilinguals, further challenging the model of the streamlined native speaker. (3) In majority language use, non-canonical patterns were dominant in spoken and/or informal registers, and this was true for monolinguals and bilinguals. In some cases, bilingual speakers were leading quantitatively. In heritage settings where the language was not part of formal schooling, we found tendencies of register leveling, presumably due to the fact that speakers had limited access to formal registers of the heritage language. Our findings thus indicate possible quantitative differences and different register distributions rather than distinct grammatical patterns in bilingual and monolingual speakers. This supports the integration of heritage speakers into the native-speaker continuum. Approaching heritage speakers from this perspective helps us to better understand the empirical data and can shed light on language variation and change in native grammars. Furthermore, our findings for monolinguals lead us to reconsider the state-of-the art on majority languages, given recurring evidence for non-canonical patterns that deviate from what has been assumed in the literature so far, and might have been attributed to bilingualism had we not included informal and spoken registers in monolinguals and bilinguals alike.

## Introduction: Heritage Speakers as Native Speakers

Heritage speakers are speakers who grow up in a bi- or multilingual home with a minority language in addition to the majority language(s) dominant in the larger society (see, e.g., Montrul and Polinsky, 2011). Accordingly, these are speakers who acquire their heritage language early and naturally in a home environment as a first language, but who also acquire another language early on, which is more dominant in the larger society and will often be the only language supported in the formal context of schooling. This constitutes an interesting population that poses a challenge for the question of what counts as a “native speaker,” which has traditionally been conceptualized mostly from a monolingual standpoint [cf. criticism in Ortega (2009) and Cook (2016)]. Investigating heritage speakers can help us unravel underlying assumptions of “nativeness,” and contribute to our understanding of native grammars, language variation and change (e.g., Polinsky, 2015, 2018; Lowe, 2020).

The concept of “native speaker” as used in linguistic research can involve at least three different types of assumptions. First, a basic assumption is the requirement that a native speaker is “native” in the sense of being born into the language, that is, acquiring it from birth. In this sense, heritage speakers are uncontroversially native speakers of their heritage language, since they acquire it as a first language in a home environment. However, this is not the only requirement for native speakers used in the literature.

A second – implicit or explicit – requirement, often found in heritage language research, is that in order to be fully recognized as such, a native speaker has to master a repertoire that also involves standard or formal registers of their heritage language. For instance, Montrul and Polinsky (2019), while explicitly acknowledging that heritage speakers “are native speakers of their heritage language” (p. 420), require a range of registers, including formal writing, for a “proficient native speaker” (p. 426; cf. also Montrul, 2008:109). Since such registers are learned primarily in the context of formal education, heritage speakers often only acquire them for the majority language, which would then exclude them from the group of proficient native speakers of their heritage language.

It is not clear, though, why specific registers should be a necessary part of native-language proficiency, since the development of register distinctions is linked to social and communicative requirements that vary across social groups. This is independent of bi- or monolingualism, and in the case of monolinguals, it usually does not affect our view of speakers as native. For instance, we would not assume that a language without formal writing does not have any proficient native speakers, for instance in historic stages before the invention of writing, or for minority languages without a written code, and we would not claim that preschool children and people who are illiterate are not native speakers of their language.

Furthermore, register knowledge may differ substantially across (monolingual) speaker groups and registers develop throughout the lifespan, so it is not clear why some registers, but not others, would be required for native-speaker proficiency. For instance, university students will typically acquire new spoken and written registers characteristic for academia, but this will not be regarded as a requirement for being a proficient native speaker, and it is uncontroversial that monolinguals without university experience are proficient native speakers of their languages.

This suggests that we ought to keep nativeness separate from register distinctions, so that speakers who use, for instance, only informal and/or spoken registers of their language, will also be considered proficient native speakers. This underlines the importance to tap into linguistic competences supporting language use in different real-life settings, including those outside standard language (cf. also Bayram et al., 2019). Avoiding a standard language bias in our research is also important given that formal standard varieties are exceptional: they are subject to codified norms that hamper normal patterns of language variation and change, and thus should be considered peripheral rather than central instances of native language grammars.

A third kind of assumption is related to attitudinal and language-ideological patterns constructing a native speaker as someone who has grown up monolingually. Heritage speakers are primarily investigated in societies with a strong monolingual habitus (Gogolin, 1994, 2002), in particular countries based on European nation state building (including those that developed out of former European settler colonies, e.g., the United States). Against this background, a “native speaker” is often taken to be monolingual [cf. criticism in Brutt-Griffler and Samimy (2001), Bonfiglio (2010), Cook (2016), and Ortega (2016)]. This conceptualizes monolinguals as the primary owners of a language, and as the gold standard for linguistic competence and attainment. Such a conceptualization was already implicit in earlier structuralist idealizations, such as the Chomskian “ideal speaker-listener” (Chomsky, 1965) or Saussure’s focus on one-to-one correlations of language and place as the “forme idéale” (de Saussure, 1916: Part 4, Ch. 2, §1). In current studies, it is implied in the use of monolinguals as a control group to test “native-like” behavior or “native competence” in heritage speakers, or to test whether some areas “develop at native levels” in heritage grammars.

However, such an idealization of the monolingual speaker as the primary bearer of a native language is not reasonable, and it is not even feasible. It is not reasonable given that multilingualism is the normal condition for human language, and, as has been amply stated (Grosjean, 1982, 2010; Romaine, 1989; Myers-Scotton, 2006, and others), most speakers in today’s world are multilingual. A focus on monolinguals as native speakers in linguistics makes as little sense as the focus on males that has lately been criticized in medical research (Criado-Perez, 2019; McGregor, 2020). Medical research has, for a long time, focused only on males because studies did not want their data to be affected by hormonal changes thought to be characteristic for female bodies – but hormonal changes are part of the human condition, and if we want to know something about humans, we have to include females. In the same vein, if we want to know something about language, we have to include multilinguals, because being linguistically multi-competent is part of the human condition when it comes to language (e.g., Cook, 2016).

A restriction to monolinguals is not even feasible for empirical research, because it is not clear who would qualify as a “true monolingual.” Language is always variable, speakers’ repertoires always involve a range of options which could be captured as different grammars (e.g., Tracy, 2002; Roeper, 2003), and the interaction of linguistic resources within repertoires is not categorically different for languages versus dialects, registers, or styles (cf. Li Wei, 2016). This suggests that there is no clear-cut distinction between bilingual speakers who use different languages and “monolinguals” whose repertoire will always include at least different registers. Furthermore, cross-linguistic effects on the L1 have even been attested for monolingual speakers who learn a second language in an instructed, non-immersion setting (Schmid and de Leeuw, 2019). Hence, if we restrict native-speaker status to monolinguals in a strict sense, then most of the worlds’ population would not count as native speakers – including most linguists today, given that most of us are fluent L2 English speakers, and thus could no longer count as native speakers of our L1.

This calls for a perspective that integrates heritage speakers into the native language continuum. Heritage language research as a field has shown that language is flexible and open to change over the lifespan. We believe that the time is ripe to take a further step and to take seriously the fact that heritage speakers are native speakers of both their languages, as emphasized in recent discussions of heritage speakers and bilingualism^1^. In what follows, we show what this means in terms of a research programme that does not take monolingual standard norms as a yardstick to identify what is missing or incorrect in heritage speakers’ language use. On the basis of findings from cross-linguistic research, we show what is to be gained by overcoming a deficit-oriented view of heritage speakers. To this end, we explore the dynamics, rather than the vulnerability, of different linguistic domains and investigate development, variation, and innovation, rather than incomplete acquisition, attrition and loss. Crucially, this means (1) that we do not concentrate on standard language and formal registers alone, but capture speakers’ broader repertoires, including informal and spoken language, and (2) that we target heritage speakers and monolinguals alike – not as test group vs. control group, but as two groups of native speakers that we expect to both show interesting patterns of language variation.

In what follows, we present results from a large-scale, cross-linguistic investigation that realized such a research programme within the context of the Research Unit ‘‘Emerging Grammars in Language-Contact-Situations: A Comparative View’ (short ‘RUEG^2^’)^3^. In our investigation, we approached bilinguals and monolinguals as two speaker groups to be investigated, rather than experimental vs. control group. Accordingly, we cast our net wide and targeted non-canonical patterns in general, that is, all patterns that would not be expected in standard grammar, and we did this for monolingual and bilingual groups alike.

This yielded a range of novel findings across languages, not only for heritage speakers, but also for the monolingual groups. In the following sections, we discuss evidence showing that a range of non-canonical phenomena in heritage speakers are also at work in monolingual speakers, pointing to language-internal tendencies of variation and change. These findings place multilinguals at the forefront of linguistic dynamics, and further support the integration of heritage speakers into the native language continuum. We have argued above that recognizing heritage speakers as native speakers is justified on conceptual and theoretical grounds. In what follows, we show that this perspective is also a better fit for the empirical data. We found a range of patterns that would be surprising if we saw monolinguals as a measure for “nativeness” and bilinguals as the deviant group. In contrast, these findings make a lot of sense if we see both groups as part of the native speaker continuum.

## Materials and Methods

The methods we used to elicit data meet the two demands formulated above: we need to include informal and spoken language, and we need to target non-canonical patterns and variation in bilinguals and monolinguals alike. In order to achieve this, we used the “Language Situations” (“LangSit”) set-up, which avoids a restriction to formal language and taps into broader repertoires across speaker groups (cf. Wiese, 2020). In this set-up, participants are familiarized with a fictional event (e.g., a car accident) and are asked to imagine themselves as a witness to this event, and then act out telling different interlocutors about it in different communicative situations. This yields naturalistic productions that are comparable across speaker groups, languages, and settings.

All materials developed for RUEG’s investigation, including stimuli, elicitor instructions, and a training video for elicitors, have been stored with the Open Science Foundation for open access at https://osf.io/cm96g/.

### Stimuli

For our investigation, we developed a video showing a (minor) car accident that involved a young woman with a dog, a couple with a baby in a pram, and two cars. In this video, one sees the couple approaching a car park, with the man bouncing a ball. Across a lane, the woman with the dog is unloading groceries from her car. The two cars are seen approaching the lane, when suddenly the man loses control of his ball, which bounces in front of the first car. On the other side, the dog gets excited and runs into the lane toward the ball, and the woman drops her groceries. The first car comes to an abrupt halt, causing the second one to bump into it. The man with the ball helps the woman pick up her groceries, the two drivers get out of their cars, and one of them calls the police, which can be seen through a close-up of the emergency number on his phone.

We developed five versions for five countries (see below): Germany, Greece, Russia, Turkey, and the United States. In order to support cross-linguistic comparisons, these versions only differed with respect to the emergency number, but were otherwise identical.

### Procedure

Participants or their parents, in the case of adolescents, gave informed consent. For the elicitation, they saw the video, were asked to imagine themselves as a witness to the accident, and then had to play-act telling different interlocutors about it. We constructed four different communicative situations by manipulating formality and mode: participants were asked to:

(1) Leave a voice message for a friend, via instant messenger (informal-spoken).
(2) Write a message to a friend, via instant messenger (informal-written).
(3) Leave a voice message on a police “witness line” (formal-spoken).
(4) Write a witness report for the police (formal-written).

For the informal language productions (1 and 2), participants used the WhatsApp© messenger on a mobile phone provided by the elicitor, where auto correction, swiping, and suggestions had been switched off. The formal-spoken message (3) was produced on the same phone, as a voice mail to the mail box of a (fictional) contact “Police Department – eyewitness line.” The formal report (4) was typed in using a simple text editor on a laptop, with spelling correction switched off.

For the different language productions, the video was shown several times. Informal versus formal productions were elicited in two different rooms that were suitably decorated according to the (in-)formality, and with two different interlocutors who acted and were dressed informally vs. formally. Short breaks filled with (in-)formal conversations divided informal and formal parts of an elicitation session.

At the end of data elicitation, participants were asked to fill in a sociolinguistic questionnaire on biographical data including language use and personality traits.

Bilingual speakers were recorded twice, in their heritage language and in the majority language, with the two sessions at least three days apart. Monolingual speakers were recorded once, in the majority language. Order of elicitation was counterbalanced for the four communicative situations, and, in the case of bilingual speakers, for the two languages.

### Participants

Participants were heritage speakers and monolingual speakers. Heritage speakers were defined as speakers who had grown up with a family language in addition to the country’s majority language. In order to participate, they had to use the heritage language regularly with at least some members of their nuclear family, and to be able to speak and write in it (although not necessarily in the standard alphabet). Further conditions were that they were born in the country of the respective majority language or had arrived there at an early age^4^ and that they had lived in that country since, although not necessarily without interruptions. Monolingual speakers were speakers who used only one language regularly at home, namely the respective country’s majority language, although they might have acquired additional languages, for instance through formal education.

The bilingual group covered heritage speakers of Greek, Russian, and Turkish in Germany and in the United States, and of German as a heritage language in the United States. The monolingual group consisted of speakers of English, German, Greek, Russian, and Turkish in the United States, Germany, Greece, Russia, and Turkey, respectively. In all categories, we covered two age groups: adolescents (14–18 years), and adults (22–35 years).

Participants had no reported speech disorders and normal or corrected-to-normal hearing and vision.

### Data Processing and Corpus Generation

Elicitations yielded matched elicited, semi-spontaneous data across registers, contact-linguistic settings, and bilingual and monolingual speaker groups, in five languages:

• German as a majority language in Germany spoken by monolingual speakers, and by bilingual speakers with Greek, Russian, or Turkish as heritage languages, and as a heritage language in the United States spoken by bilingual speakers with English as a majority language;
• English as a majority language in the United States spoken by monolingual speakers, and by bilingual speakers with German, Greek, Russian, or Turkish as heritage languages;
• Greek, Russian, and Turkish as majority languages spoken by monolingual speakers in Greece, Russia, or Turkey, respectively, and as heritage languages spoken by bilingual speakers with English or German as majority languages in the United States or Germany, respectively.

In what follows, we refer to languages spoken as majority languages, e.g., German in Germany, or Greek in Greece, as “majority German/Greek” or short “maj-German/- Greek,” etc., and to languages spoken as minority languages in a heritage context, e.g., German or Greek in the United States, as “heritage German/Greek” or short “h-German/-Greek,” etc. We will use “HS” as an abbreviation for “heritage speaker.” When we give examples, we provide the transcriptions for spoken data, and keep to the original spelling (including possible typos) in the case of written data.

Codes identifying data from the RUEG corpus provide the following information, in this order:

• Country: DE – Germany; GR – Greece; RU – Russia; TU – Turkey, and US – USA.
• Bi-/monolingual speaker: bi vs. mo.
• Speaker number incl. age group: 1–50 – adults; from 51 onward – adolescents.
• Gender: M vs. F (there were no speakers who identified as non-binary).
• Heritage language for bilingual speakers or only family language for monolinguals: D – German; E – English; G – Greek; R – Russian; and T – Turkish.
• Communicative situation: f – formal/i – informal and s – spoken/w – written.
• Language of production: D, E, G, R, and T.

For instance, “DEbi51MT_isD” identifies data in Germany (DE) from a bilingual (bi) adolescent (51) male (M) speaker with Turkish (T) as a h-language in an informal (i) spoken (s) setting, communicating in German (D).

All corpus data has been anonymized and integrated into a unified corpus, the RUEG corpus (Wiese et al., 2019). The RUEG corpus is a multimodal and multi-layer corpus, which in its current version (0.4.0) contains approximately 520,100 tokens^5^ (appr. 146,000 for English, 157,000 for German, 66,000 for Greek, 88,000 for Russian, and 63,000 for Turkish), based on data from 716 speakers, of whom 393 are bilingual and 323 monolingual. Table 1 gives the details for the different data sets^6^.

**TABLE 1:** RUEG corpus data.

Country	Bi-/monolingual	Languages	# speakers	# tokens
DE	Bilingual	maj-German	44	21,339
		h-Greek	47	19,783
	Bilingual	maj-German	56	34,503
		h-Russian	58	32,882
	Bilingual	maj-German	65	35,881
		h-Turkish	65	23,722
	Monolingual	maj-German	64	50,706
United States	Bilingual	maj-English	34	16,765
		h-German	34	14,888
	Bilingual	maj-English	64	30,913
		h-Greek	64	18,032
	Bilingual	maj-English	65	36,021
		h-Russian	66	29,214
	Bilingual	maj-English	59	32,905
		h-Turkish	56	18,502
	Monolingual	maj-English	64	29,238
GR	Monolingual	maj-Greek	64	27,931
RU	Monolingual	maj-Russian	67	25,930
TU	Monolingual	maj-Turkish	64	20,947

At the time of writing, the corpus continues to grow, with more data sets and improved annotations added. Corpus data includes language productions in all four communicative situations, with additional transcriptions for spoken data (conditions 1 and 3), and the biographical data from the speaker questionnaires. Language productions are annotated for syntactic spans, lemmata, language, and parts of speech in a universal and a language-specific set of categories. The corpus can be used via the ANNIS corpus search and visualization tool (Krause, 2019). The complete corpus, including its source data and all preliminary versions, is freely available in an open repository (10.5281/zenodo.3236068).

## Results

Our analyses yield three main findings: (1) cross-linguistically, we find non-canonical patterns not only in heritage speakers, but also in monolinguals, including patterns that, according to the literature, would not be expected for monolinguals; (2) we find extensive variation not only in heritage speakers, but also in monolinguals; (3) non-canonical patterns interact with register, underlining the importance of taking into account both formal and informal settings and, crucially, doing so for multilinguals and monolinguals alike.

### Non-canonical Patterns: Not Just in Heritage Speakers, but Also in Monolinguals

In order to demonstrate what can be gained by approaching heritage speakers as native speakers of both their languages, we present non-canonical patterns that we observed in both heritage speakers and monolinguals. These are patterns that have so far been considered absent from native grammars and which might have been attributed to bilingualism had we taken a less inclusive approach. We cover domains of morphology and syntax, intonation, and pragmatics. In what follows, we present results from different languages, combining, in each case, qualitative and quantitative analyses. Qualitative analyses capture the relevant patterns and their distribution across bilingual and monolingual speaker groups. Quantitative analyses compare frequencies between different groups. In domains where corpus frequencies are high enough, this is supported by statistical tests^7^. For lower-frequency phenomena, we provide comparative figures for the different groups through relative (rather than absolute) numbers for non-canonical cases as a proportion of all relevant cases.

#### Morphology and Syntax

In the domain of morphology and syntax, examples come from the formation and use of participles in Russian, word order in German, and bare NPs in German.

##### Non-canonical Participles in Russian

Participles in Russian are challenging in their morphology and syntax, and they are acquired later by monolinguals (Cejtlin, 2009; Tribushinina et al., 2013), which makes them an ideal domain to look for non-canonical forms. Results of our corpus study show that morphologically non-canonical participles can be found across all speaker groups, including monolinguals:


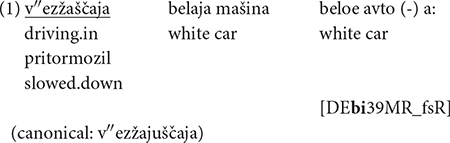


“A white car is driving in, the white car (-) a: slowed down.”


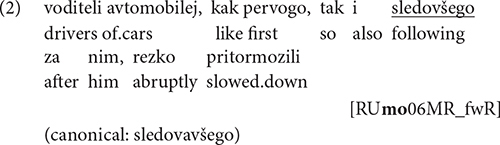


“The drivers of the cars, both the first one and the one that followed it, abruptly slowed down”

A qualitative analysis reveals interesting dynamics in the morphological formation of participles. With suffixes, there is a widespread truncation of material, which can be seen both in (1), produced by a HS, and (2), produced by a monolingual. In (1), the expected suffix -*jušč*- for the formation of active present participles of open stems ending with a *j*-addition (Bogdanov et al., 2009) is truncated to -*šč*-. In (2), the base for the formation of the active past participle with the suffix -*vš*-, consisting of the stem and a thematic suffix *sled*-*ova*-“follow,” is truncated to *sled-o*-. Such a pattern can be interpreted as a reduction of morphological complexity pointing at a tendency for stem unification across paradigms (Gagarina, 2002:160).

**FIGURE 1:**
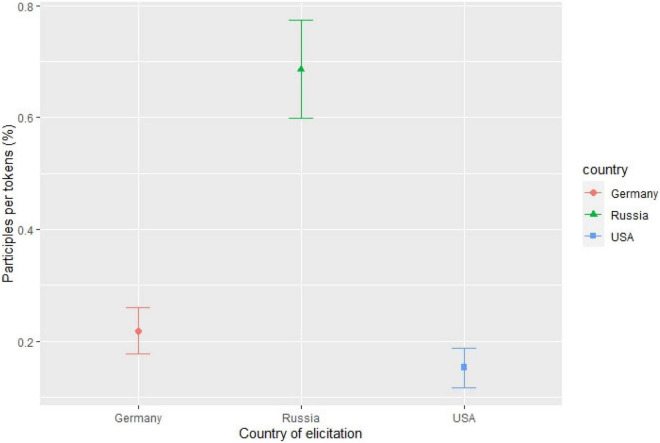
Relative frequency of participles per tokens (%) across different groups.

In order to check whether the frequencies of participles differ significantly across groups, we ran a one-tailed unpaired Wilcoxon rank sum test. Results show that HSs produced more non-canonical forms than monolinguals (*M* = 0.96, *SE* = 0.48): *W* = 36,480, *p* = 0.023 for HSs in the United States (*M* = 4.64, *SE* = 1.29), and *W* = 31,997, *p* = 0.032 for HSs in Germany (*M* = 4.11, *SE* = 1.28). Overall, HSs produced fewer participles, both canonical and non-canonical ones, than monolinguals (*M* = 0.69, *SE* = 0.09): *W* = 28,455, *p* < 0.001 for HSs in the United States (*M* = 0.15, *SE* = 0.04), and *W* = 26,118, *p* < 0.001 for HSs in Germany (*M* = 0.22, *SE* = 0.04), see Figure 1 for the relative frequencies and Table 2 for absolute frequencies of tokens and participles across the groups. Both findings might be explained by the abovementioned status of participles, and by the fact that they are generally rare in HSs’ oral input, since they are associated with formal registers (Zemskaja, 1973; Golub, 2001).

**TABLE 2:** Frequency of tokens and participles across different groups.

Country	Tokens: overall	Tokens: canonical participles (% of all tokens)	Tokens: non-canonical participles (% of all tokens)
DE	32,882	57 (0.17%)	14 (0.04%)
US	29,214	40 (0.13%)	14 (0.05%)
RU	25,930	151 (0.58%)	6 (0.02%)

Non-canonical participle formation is also well documented for monolingual child acquisition (Cejtlin, 2009). Interestingly, such forms often follow the same patterns as those we find in our data:


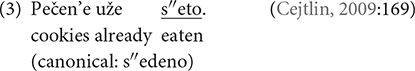


“The cookies are already eaten”

Similar to (1) and (2), example (3) is a case of morphological truncation of a stem *s″ed-* “eat”- to *s″e-*, which then forms the base for participle formation with the suffix *-t-* rather than with canonical -*en*(*n*)*-*. The fact that we observed such patterns also in monolingual and bilingual (HS) adult speakers, suggests ongoing internal dynamics in this linguistic domain in Russian. That HSs use such a pattern with a higher frequency hence means that they can shed a spotlight on ongoing tendencies in native grammars.

##### Non-canonical Word Order in German

For German, we report relevant findings from two domains: word order and bare NPs. German has traditionally been described as an SOV language with verb-second (V2) word order requiring the finite verb in main declaratives to appear in second position, after exactly one constituent in the domain in front of it, the “forefield.” This position of the finite verb constitutes one of two “sentence brackets” characteristic for the lay-out of German sentences. The other position is located at the right clausal periphery. In main declaratives, it contains non-finite verbs and separable verb particles. Together, the left and the right bracket delimit the “middle field,” the canonical domain for complements and adjuncts. Typically, embedded clauses are extraposed, i.e., occur beyond the right sentential bracket in the “post-field.”

The V2 requirement is usually regarded as a prime example of a rigid constraint in the grammar of German native speakers. Therefore, deviations from V2 in the maj-German of Turkish HSs, where an adverbial occurs in front of the subject at the left periphery, were taken to fall outside native German. For Auer (2013:37f), for instance, they indicated the reorganization of German V2 to SVO, which would “intervene deeply in the structures of autochthonous German in its standard and non-standard forms” and, together with other non-canonical patterns, such as bare NPs, “would have the potential to constitute a new variety that would differ substantially from autochthonous German” (German originals, our translation).

This view has been challenged by accounts integrating this non-canonical pattern into the syntactic lay-out of German sentences (Wiese, 2013; te Velde, 2017; Walkden, 2017; Wiese and Müller, 2018). Findings suggest a systematic verb-third (V3) option that, unlike SVO, preserves the characteristic German sentence brackets. From the point of view of information structure, V3 has the advantage over V2 of allowing both a framesetter or discourse linker [e.g., *dann* “then,” see (4) and (5) below] and a topic in the left periphery (Wiese, 2012, 2013; Walkden, 2017; Wiese et al., 2017; Bunk, 2020).

While V3 in German has mostly been associated with language-contact situations (e.g., Walkden, 2017), we have shown that it is also available in monolingual speakers (Wiese and Rehbein, 2015; Bunk, 2020). The present study confirms this for maj-German across populations: in the RUEG corpus, we find V3 not only in bilingual speakers with h-Greek, h-Russian, and h-Turkish, but also in monolingual speakers, cf. (4) and (5).


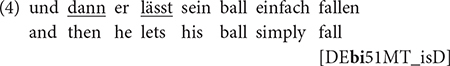


“And then, he just lets his ball fall down.”


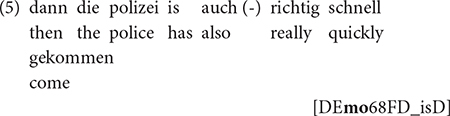


“And then, the police arrived really quickly.”

Findings point to the same V3 options in monolinguals and bilinguals, with an adverbial and a subject preceding the finite verb. As in previous studies, V3 is infrequent, though, with only 48 cases in the bilingual and 11 cases in the monolingual group, with the bilingual group in the lead quantitatively (cf. also Wiese and Rehbein, 2015). In order to compare the difference, we computed normalized frequencies per 100 CUs (communicative units). In the RUEG corpus, CUs were used as a means to segment utterances and were defined as an “independent clause with its modifiers” (following Loban, 1976:9). Accordingly, normalizing for 100 CUs gives us the numbers for V3 as a percentage of all independent clauses, and hence a good approximation for the proportion of non-canonical cases, since V3 is a pattern located at the clausal level. The quantitative difference between speaker groups we observed for absolute numbers is confirmed for such normalized frequencies: we find 0.96 V3 occurrences per 100 CUs in the bilingual group, compared to 0.41 occurrences per 100 CUs in the monolingual group.

Interestingly, the higher frequency is primarily due to speakers with h-Turkish: data from this group makes up 38 of the 48 V3 findings, or 1.88 occurrences per 100 CUs, compared to only 0.42 and 0.27 occurrences per 100 CUs (five cases each) coming from the h-Greek and h-Russian group, respectively. These findings speak against contact-linguistic transfer, since Turkish, an SOV language, would support the basic SOV word order of German, while Russian and Greek both have a tendency to SVO, which shares a strong surface similarity with V3 as soon as additional left-peripheral constituents such as adverbials are involved. If cross-linguistic transfer were a relevant trigger for V3, we would expect bilingual speakers with h-Greek and h-Russian to be in the lead, rather than those with h-Turkish. This further supports a view of German grammar as the locus of this phenomenon.

For h-German speakers in the United States, we observe an increase in the non-canonical V3 pattern, similar to what we found for non-canonical participle formation in h-Russian: h-German speakers produce 3.47 V3 main clauses per 100 CUs (55 cases in total), with framesetters/linkers like *dann*, *nun* in addition to those probably adopted from English, such as *so* ([zo]), cf. (6) and (7) (with the latter exhibiting all three linkers).


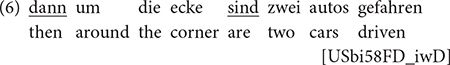


“then two cars drove around the corner”


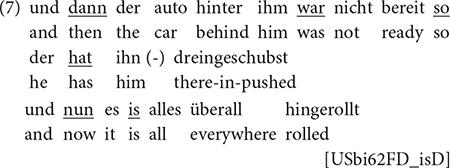


“and then the car behind it wasn’t ready so it pushed into it and now everything rolled everywhere”

In this case, the influence of English (X)SVO might further support V3 production, given that English is the majority and main contact language for h-German here. While we cannot rule out that some patterns are enhanced by parallels in English, this only holds partially, though, as shown in (6) and (7), where the clausal brackets remain canonical (“sind … gefahren;” “hat … dreingeschubst”), in contrast to English SVO. Qualitatively, the h-German data matches, to a large extent, what we find in monolingual and bilingual speakers of maj-German. The difference appears to be quantitative and due to an increased range of constituents involved in V3 clauses, such as non-subjects in the forefield [e.g., a PP as in (6)] and – trivially – borrowed linkers [such as *so* in (7)]. All other cases are attested in our monolingual data as well, even though with a lower frequency.

Had we only investigated h-German in contact with maj-English, we might have claimed that V3 is due to cross-linguistic transfer. Instead, we can now conclude these patterns are also available within the monolingual German repertoire but may be selectively strengthened in HSs by language contact.

##### Non-canonical Bare NPs in German

For the investigation of non-canonical bare NPs in German, we used additional data from the DNam corpus of German in Namibia (Wiese et al., 2017; Zimmer et al., 2020)^8^, in order to compare two groups of h-German speakers. Namibian German represents a rare case of h-German that is still grounded in a vital speech community that systematically uses German not only in informal, but also in formal settings. We focus on the LangSit subcorpus which contains register-differentiated data similar to that in the RUEG corpus (103 speakers; 51,509 tokens). It covers informal-spoken and formal-spoken productions (formal: 23,606 tokens, informal: 25,629 tokens), elicited with visual stimuli in the form of a photo story about a car accident.

Our findings indicate that monolinguals as well as bilinguals produced non-canonical bare NPs, i.e., those that we would not expect in standard German. Figures are overall low: non-canonical cases make up 0.34% of all NPs across maj-German data by both monolingual and bilingual speakers in Germany (22 and 50 occurrences, respectively), 0.98% (22 cases) in h-German in the United States, and 1.06% (44 occurrences) in Namibia.

Interestingly, the non-canonical cases differ qualitatively between the h-German group in the United States and the others. Non-canonical NPs in Germany (maj-German by mono- and bilingual speakers) and Namibia (h-German) can be subsumed under current trends of article decline in German triggered by hyperdetermination, as described by Leiss (2010): (a) generally in generic and unique reference and light verb constructions and in local and directional contexts, and (b) a decline of the definite article in initial, thematic position, and of the indefinite article in rhematic position, because these are already inherently definite or indefinite, respectively. (8) illustrates this for a non-canonical bare NP with generic reference (and in rhematic position) from Namibian German, and (9) for one in rhematic position, produced by a monolingual speaker in Germany:


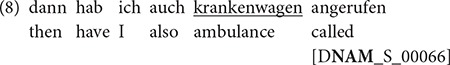


“Then, I also called (an) ambulance.”


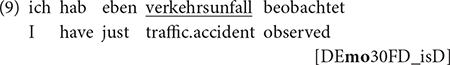


“I just observed (an) accident.”

The h-German data in the United States differs from this in that we find a distinctive pattern that accounts for almost half of the cases (11 occurrences) and does not occur in the other data. In this pattern, non-canonical bare NPs form the second element in a coordination, cf. (10):


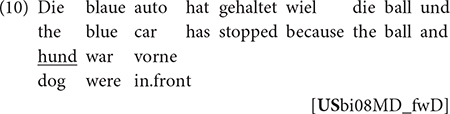


“The blue car stopped because there was the ball and (the) dog in front of it.”

Unlike known patterns of determiner sharing (McCawley, 1993; Ackema and Szendrõi, 2002), this is not restricted to coordination at the VP level, suggesting that an existing pattern can be further extended in heritage language contexts. As the contrast to Namibian German shows, this variation is not related to heritage German *per se*, but might differ across speech communities. Hence, heritage languages can participate in ongoing tendencies as well as further extend native-grammar options.

#### Intonation

For the domain of intonation, our data provides an example from Russian yes-no questions (YNQs). In the literature, Russian YNQs are reported to be realized with a bitonal rising nuclear pitch accent on the verb (L* + H or L + H*; Rathcke, 2006b; Meyer and Mleinek, 2006) followed by a low final boundary tone (FBT; L%) (Igarashi, 2006; Rathcke, 2009), except if the nuclear pitch accent falls on the final syllable, in which case a high FBT (H%) is realized (Makarova, 2003; Rathcke, 2006a). The FBT can thus be considered truncated if no material follows the pitch accent (Rathcke, 2009, 2013). The intonation patterns of YNQ differ in Russian and English and are hence interesting to investigate in bilingual speakers.

In order to study the prosodic realization of YNQs of mono- and h-Russian speakers, we elicited experimental data in addition to the corpus data during data collection. This consisted of 10 read-aloud YNQs about details of the car accident. We recorded 20 speakers per group, i.e., (1) bilingual speakers of h-Russian in the United States, (2) bilingual speakers of h-Russian in Germany, and (3) monolingual speakers in Russia [see Zuban et al. (2020) and in prep. for details].

The elicited YNQs differed in the number of syllables following the nuclear pitch accent (or an additional pitch accent on the object for SVO questions). Each YNQ was annotated for the location of the nuclear pitch accent and FBT, following a combined phonetic and auditory approach: presence of a pitch accent was detected auditorily, and the FBT was examined with respect to local F0 trajectories and changes using Praat. Labeling followed the autosegmental-metrical framework (Makarova, 2003; Igarashi, 2006; Rathcke, 2006b).

**FIGURE 2:**
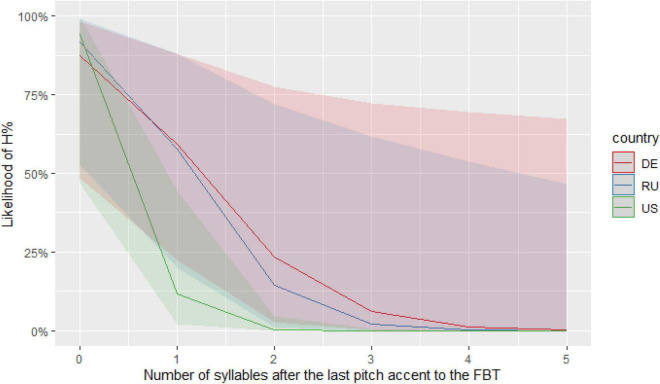
Estimated likelihood of H% being chosen over L% based on the productions of the three speaker groups.

Results of the descriptive analysis showed that h-Russian speakers in the United States predominantly produced L% (82% of all cases) while h-Russian speakers in Germany and monolinguals produced L% less frequently (58% for both groups). In order to check for a possible impact of multiple fixed effects on the distribution of high and low boundary tones, we ran a binomial generalized linear mixed-effects model with FBT as the dependent variable, and the three speaker groups, number of syllables following the last pitch accent to the FBT (0–5), transitivity, nuclear contour as independent variables, and with speaker and item as random effects (Figure 2; see “Supplementary Appendix 1” for full model specifications and summaries). It was found (among other things) that all speaker groups produced an H% when the last pitch accent fell on the final syllable, in line with the literature on Russian. Along the same lines, according to what was reported on truncation of FBTs in Standard Russian, h-Russian speakers in the United States chose the L% FBT as soon as there was at least one syllable following the last pitch accent. However, h-Russian speakers in Germany and mono-Russian speakers preferred the L% only when there were more syllables following the last pitch accent.

Hence it is only h-Russian speakers in the United States who behave according to what was described in the literature, while mono- and h-Russian speakers in Germany do not. Had we investigated only h-Russian in Germany and found the significantly increased use of H% (i.e., the absence of categorical truncation), we might have been led to think that this pattern is a specific feature of HSs’ intonation grammar, possibly due to influence from maj-German, which has H% in YNQs (Grice et al., 2005). However, comparable realizations by mono-Russian speakers show that what we see here is a more general pattern: if we approach both HSs and monolinguals as native speakers and analyze language use across speaker groups, we may also find non-canonical patterns in monolinguals that might otherwise have been attributed to bilingualism.

#### Pragmatics

In the domain of pragmatics, examples come from data on the position of new referents in Turkish and on referent introduction in English.

##### Non-canonical Placement of New Referents in Turkish

As briefly mentioned in “Morphology and Syntax,” the basic and pragmatically neutral word order in Turkish is SOV. However, for specific pragmatic purposes, elements can also be placed in the post-verbal position. In particular, this is possible for marking backgrounded information and afterthoughts (Erguvanli, 1984; Schroeder, 1995; Kornfilt, 1997), for constituents such as NPs, adverbs, discourse markers, forms of address, and finite subordinate clauses. What is crucial is that placement of new information is believed to be impossible here: according to the literature, the information placed in the post-verbal position has to be discourse-predictable or recoverable from previous discourse (Erguvanli, 1984:56).

Approaching HSs as native speakers of their languages, we investigated these information-structural restrictions for h-Turkish and mono-Turkish alike. We proceeded by selecting the 21 most frequently used nominal referents that played a role in the elicited narratives. We annotated every occurrence of these, adapting Riester and Baumann’s (2017) referent annotation scheme, and identified information status through three categories (cf. Schroeder et al., to appear): (1) “new” (first mention), (2) “given” (referent that was introduced before) (3) “bridging” [referent that has not explicitly been introduced but belongs to the “pragmatic set” in the sense of Hawkins (1984) of a given referent (anchor)]. Afterthoughts, repairs and finite subordinate clauses were excluded from analysis.

Results of the analysis show that even though most of the referents placed post-verbally were indeed “given” and “bridging,” there is a substantial number of new referents used in the post-verbal position. What is interesting in the context of the present manuscript, is that new referents in the post-verbal position occurred not only in the data of HSs [as seen in (11), but also in monolinguals in Turkey (12)]. In HSs in Germany, the occurrences of new referents in post-verbal position constitute 32.43% of the overall number of referents in the post-verbal position (24 new referents out of 74 referents in the post-verbal position), in monolinguals in Turkey the occurrences of new referents constitute 22.45% (11 new ones out of 49 referents), and in HSs in the United States, the new referents in the post-verbal position make up 21.43% (24 occurrences out of 112). This outcome contradicts what the literature says about canonical Turkish, namely that new referents are not possible in the post-verbal position.


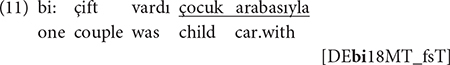


“There was a couple with a stroller.”


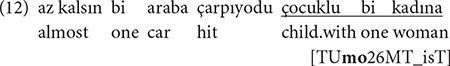


“A car almost hit a woman with a child.”

Most of the new referents in post-verbal position stand in a close semantic relationship to the subject of the clause. This relationship is wider than the “bridging” relation, and it is often indicated by means of the possessive suffix *-*(*s*)*I* on the post-verbal constituent [like in (11)], or by free adjuncts that carry adverbial case. Less often, a new referent in post-verbal position is a lexical subject or object [as in (12)].

However, it would be misleading to call those new referents that are in a close semantic relationship with the subject “backgrounded information,” since in about half of the cases in our data, the referent introduced in the post-verbal position is mentioned again in the subsequent discourse, and is treated as “given” when mentioned again. We propose to call such referents “secondary” new referents, in the sense that they are secondary (and related) to another new referent with a higher relevance to the discourse at that point.

Thus, we conclude that it is indeed possible to place new information in post-verbal position in Turkish, and this is not a feature that is typical only for HSs, since the pattern is also found in the monolingual data from Turkey. As we will discuss in more detail in section “Register Leveling in Heritage Languages,” in monolinguals this pattern seems to be associated with informal registers. Hence, if we compare like with like and systematically include data from such registers from monolinguals as well, we can avoid misattributing some non-canonical patterns to bilingualism that form a more general part of native grammars.

##### Non-canonical Referent Introduction in English

Unlike Turkish, English marks newness and givenness of referents through indefinite and definite articles (Hickmann and Hendriks, 1999). The indefinite article *a* presupposes that the referent of the NP is new and the addressee is not familiar with it. The definite article *the* implies that the addressee can uniquely identify the given referent of the NP based on previous discourse, shared physical environment and/or general world knowledge (Payne and Huddleston, 2002:368–371).

Previous research has shown that bilingual speakers often differ in their production of articles from monolingual speakers of English. For example, child HSs of other languages have been reported to oversupply *the* in indefinite contexts and *a* in definite contexts in maj-English, regardless of their heritage language (Zdorenko and Paradis, 2008, 2012). Further, adult L2 English speakers with article-less L1s tend to overuse *a* and *the* in contexts with mismatching parameters of definiteness and specificity (i.e., subjective noteworthiness of the referent to the speaker) (Ionin, 2006; Ionin et al., 2008).

Most importantly, many studies on article use compare only two or more bilingual groups to each other (Lardiere et al., 2004; Zdorenko and Paradis, 2008, 2012; Ionin and Díez-Bedmar, 2021). If a monolingual comparison group is added, monolinguals usually supply articles in strict accordance with the expectations based on the literature (Hawkins et al., 2006; Ionin, 2006; Ionin et al., 2008; Sarko, 2009; Snape et al., 2013). Overall, these studies seem to suggest that variability in article production is a result of bilingualism.

We tested this assumption by examining article choice in new and given referents among bilingual and monolingual English speakers of maj-English -- 214 HSs with various heritage languages and 64 English monolinguals.^9^ Similarly to the investigation of new postverbal referents in Turkish just discussed, we selected 19 frequent referents such as *man*, *dog*, *car1*, and *car2*, and coded them for their information status as “new” (the first mention of an entity without any identifying information) or “given” (all the subsequent mentions) (Riester and Baumann, 2017). This yielded 4,961 new and 10,881 given referents.

We identified all new and given referents that were part of unexpected non-canonical structures, that is, “*the* + new” and “*a* + given” referents. All other structures in which referents appeared, including expected canonical structures (“*a* + new” and “*the* + given”), were marked as “other.” Contrary to what would be expected from the literature, we found non-canonical patterns not only in bilinguals’ productions [see (13) and (15)], but also in those of monolinguals [see (14) and (16)], and we found this for both the “*the* + new” pattern [(13) and (14)] and the “*a* + given” pattern [(15) and (16)]:


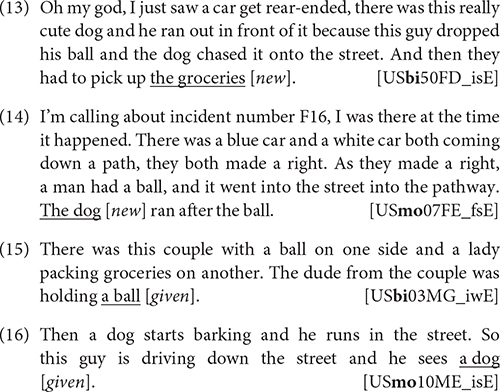


Hence, we did not find any qualitative differences between monolinguals and bilinguals in this domain. In order to check for a possible impact of bilingualism on quantitative distributions while taking into account inter-individual speaker variation, we ran two binomial generalized linear mixed effects models, one for new and one for given referents. The dependent variable was Determiner (“*the* + new”/“*a* + given” vs. “other”) and the independent variables were Bilingualism (bilingual vs. monolingual), Setting (formal vs. informal) and Mode (spoken vs. written) (see “Supplementary Appendix 2” for full model specifications and summaries).

The results indicate neither a main effect of bilingualism, nor its interactions with other variables, meaning that we have no evidence of differences between HSs and monolinguals. Both groups produced similar numbers of non-canonical “*the* + new” referents, ranging from 6.6% of all given referents by heritage speakers and 5.6% by monolinguals in the formal written situation, to 9.1% by heritage speakers and 11.1% by monolinguals in the informal spoken situation. The two groups did not differ in the production of “*a* + given” referents either: for this pattern, the percentages of non-canonical referents were much smaller and ranged from 0.47% of all new referents by heritage speakers and a complete absence by monolinguals in the informal written situation, to 0.75% in the informal spoken situation by heritage speakers and 1.5% by monolinguals. Overall, our data shows that a pattern that has mostly been attributed to bilingualism actually manifests itself in the productions of English monolinguals in the same way as it does in the speech of bilinguals.

### Variation: Not Just in Heritage Speakers, but Also in Monolinguals

In the literature, HSs and bilingual speakers in general are often presumed to exhibit higher degrees of variation than “regular” native speakers. Seton and Schmid (2016:341) even claim that “[t]he most striking characteristic that sets bilinguals apart from monolinguals is a larger amount of variability in performance.” Observations from our data suggest a more complex picture and point to intricate dynamics between groups and individual effects and different degrees of dynamicity in linguistic subsystems. The evidence provided to illustrate this point here comes from our corpus data on maj-German, spoken by monolingual and bilingual speakers in Germany (see Table 1 above).

**FIGURE 3:**
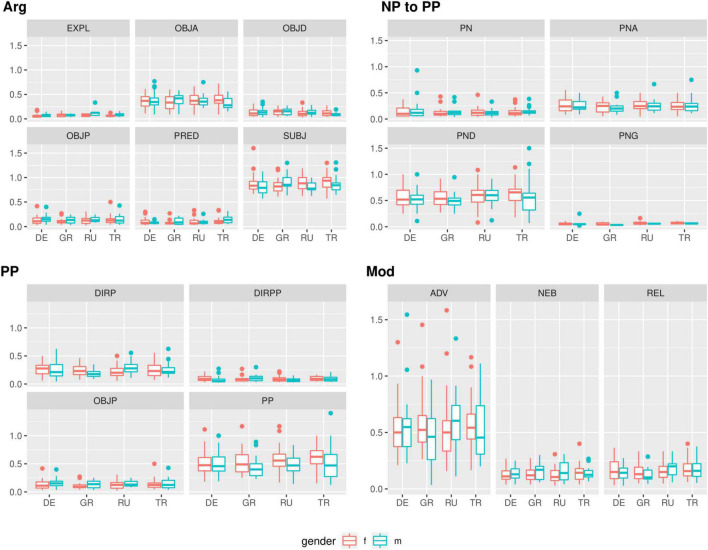
Dependencies in maj-German (formal-written) RUEG texts, normalized by lexical verbs.

Variation can be measured in different quantities, or in different qualities, such as a wider range of structures. For example, a bilingual speaker may use different structures from a monolingual, and those may be canonical or non-canonical (some canonical structures may be dispreferred by some speakers and/or in some registers). In our data, we find that all syntactic dependencies, such as different types of objects, particles, modifiers, etc., are used by mono- and bilinguals alike (see Figure 3). Hence, we do not find qualitative differences here: bilinguals (as a group) neither avoid certain dependencies used by monolinguals, nor do they exhibit a wider range of dependencies than monolinguals^10^.

Are there quantitative differences in variability, i.e., differences in variance, then? The picture here is more mixed. First of all, both the mono- and the bilingual speakers show large degrees of quantitative variance for some, but not all structures. This is in part an effect of the general frequency of a structure: if a dependency type is overall rare, its frequency of occurrence will exhibit floor effects. However, it also appears that some structures, whether rare or more frequent, are subject to more free choice in the frequency of their realization. This can be due to simple causes, such as the possibility to name all vs. just some of the agents or circumstances in the respective narratives, or recurrence and repetition of previously named entities vs. ellipsis. At the same time, a more or less frequent realization of elements may also be indicative of differences in communicative style (explicitness, emphasis) or formality (assumption of interlocutor expectations; see also Ahern et al., 2019: 487, 488). This can, for example, affect the frequency of modifying structures such as adverbs or relative clauses, which can be used to specify or comment on another dependency. Explicitness, referring here to the tendency to explicitly name more aspects of the environment or circumstances, may also play a role for different amounts of PP realizations.

However, the realization of PPs can also be due to interactions with the lexicon, for example in the case of prepositional objects required by certain verbs (abbreviated as OBJP – a full list of abbreviations is provided in “Supplementary Appendix 3”). Other PPs can be added attributively without lexical constraints. Differences in the frequency of realization of nominal complements to prepositions (PND for dative objects to prepositions, PNA for accusative objects complementing prepositions) can thus be due to lexical diversity (different verbs requiring different complements), but might also point toward case dynamics (identical verbs occurring with different complement cases). Differences in variance can thus represent very different types of phenomena.

In our data, we see variable degrees of variance between structures, but there is barely any evidence for a higher degree of variability in bilinguals. Only the h-Turkish speakers appear to show a higher degree of variance in the realization of (free) prepositional phrases (PP) and dative complements to prepositional phrases (PND), and the effect is rather small. Some bilingual groups appear to show a slightly higher variance in modifying structures (male h-Russian and male h-Greek for subordinate clauses, marked NEB, male h-Greek and male h-Turkish for adverbs, ADV). However, some chance results are to be expected due to the high number of between-group comparisons.

Overall, there is no clear trend toward higher variability in bilinguals: mono-German speakers are generally within the range of variance found for bilingual speakers, and even have a tendency to be on the upper end of it. In fact, differences between gender groups are generally higher than between speaker groups of different language background but the same gender.

**FIGURE 4:**
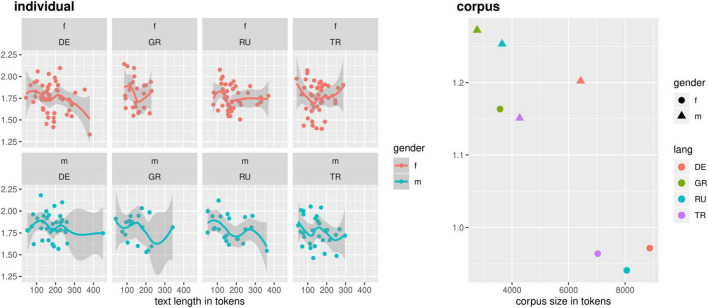
Transformed TTR in maj-German (formal-written) RUEG texts.

This also appears to be the case for lexical richness, approximated here with a transformed type-token-ratio (TTR, see Figure 4)^11^. We find no strong differences in the distribution of individual speakers in each language group. However, the female mono-German and female h-Turkish bilinguals show the highest degrees of variance in TTR. This may be an artifact of different group sizes (23 female, 10 male) -- especially since, in general, we find higher variance in the male groups. This becomes particularly obvious in the distribution by subcorpora: we find much higher TTRs for the male speaker groups compared to the female ones *across language groups*. Male and female speakers use roughly the same amount of lexemes per speaker, but those lexemes converge less between male than female speakers. This suggests divergent effects of idiomaticity or coselectional constraints^12^ and might be attributed to communicative style or a higher degree of adjustment to assumptions of interlocutor expectations.

Overall, we find different degrees of variance in our data, but no particular effect for monolinguals vs. bilinguals. In fact, the monolingual speakers in the RUEG corpus exhibit a degree of variance that reaches, and sometimes surpasses, that of the bilingual speakers. We do not find evidence that variability in the realization of syntactic structures or in lexical richness could be used as a criterion for the definition of a “real” native speaker that would favor monolingual over bilingual speakers.

### The Role of Registers

Approaching HSs as native speakers of both their languages, we targeted formal and informal registers in bilinguals and monolinguals alike. Our findings indicate an important role of registers for non-canonical patterns, and this can play out differently in heritage and majority language use. Below we discuss evidence from English and German as majority languages (“Noncanonical Phenomena in Informal or Spoken Registers of Majority Languages”), and German, Turkish, and Greek as heritage languages (“Register Leveling in Heritage Languages”).

#### Non-canonical Phenomena in Informal or Spoken Registers of Majority Languages

In majority language use, non-canonical patterns are dominantly found in informal and/or spoken registers, and this holds across bilingual and monolingual speaker groups.

##### Association With Spoken Registers for Non-canonical Patterns in English

In section “Pragmatics” we discussed two non-canonical patterns involving new and given referents in maj-English, namely “*a* + given” and “*the* + new” referents, which appear in bilingual and monolingual English speakers alike. Our further results reveal the influence of spoken vs. written mode on non-canonical article choice. In the two linear mixed effects models reported in section “Pragmatics,” we observed a main effect of mode across speaker groups and formal/informal settings: the spoken mode showed more non-canonical structures than the written mode (for “*the* + new”: mean_formal spoken_ = 7.68%, mean_formal written_ = 6.12%, mean_informal spoken_ = 10.1%, mean_informal written_ = 7.3%; for “*a* + given”: mean_formal spoken_ = 0.69%, mean_formal written_ = 0.57%, mean_informal spoken_ = 1.11%, mean_informal written_ = 0.23%; see “Supplementary Appendix 2” for *p*-values and model summaries).

The higher variability in article choice in the spoken mode could be associated with a higher cognitive load of online (spoken) productions compared to offline (written) ones. Spontaneous spoken production often exerts performance pressure since it allows little time for planning and no possibility for changing what has been said (Pullum and Huddleston, 2002:12). This factor is important in L2 research: for instance, Ionin et al. (2021) argue for testing article knowledge of L2 speakers in comprehension rather than production in order to avoid the performance pressure and to evaluate speakers’ implicit sensitivity to (in)definiteness. In our study, performance pressure in the spoken mode might have led the speakers to only consider their own perspective (familiarity with the referent) and, consequently, use the definite article, while ignoring the addressee’s perspective (unfamiliarity with a new referent), which would require the indefinite article.

In addition, we might be witnessing a new development in English: possibly, the definiteness distinction is becoming less strict in spoken spontaneous productions. So far, it is unclear if this is a systematic pattern of English internal dynamics (since over 90% of *the* uses are still canonical), and it needs to be confirmed in future research.

##### Association With Informal Registers for Non-canonical Patterns in German

In maj-German, non-canonical V3 (as described in “Morphology and Syntax” above) occurs mainly in the informal productions in our corpus data: of 59 V3 cases in maj-German altogether, 53, that is, roughly 90%, are from informal communicative situations, and this pattern is also evident for normalized frequencies, with 1.24 of 1.37 occurrences per 100 CUs, that is, roughly 91%, from informal communicative situations.

Within the informal settings, we find V3 across spoken (17) and written (18) modes. This suggests that unlike non-canonical article choice in maj-English, V3 in maj-German is primarily associated with informality, independently of mode.


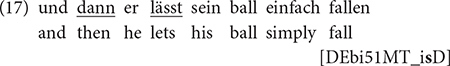


“And then, he just lets his ball fall down.”


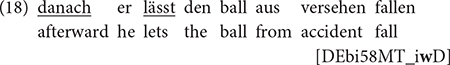


“Afterward, he accidentally lets the ball fall down.”

Note, though, that we do find a few occurrences of V3 in formal data, and this also includes one production (formal-written) from a monolingual speaker:


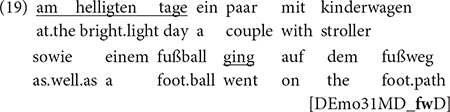


“In bright daylight, a couple with a stroller went along the sidewalk.”

This, again, integrates bilingual and monolingual speakers alike into the native speaker group: we find the non-canonical V3 pattern across groups, in both groups primarily associated with informal registers, with some exceptions which are also evident in both groups.

**FIGURE 5:**
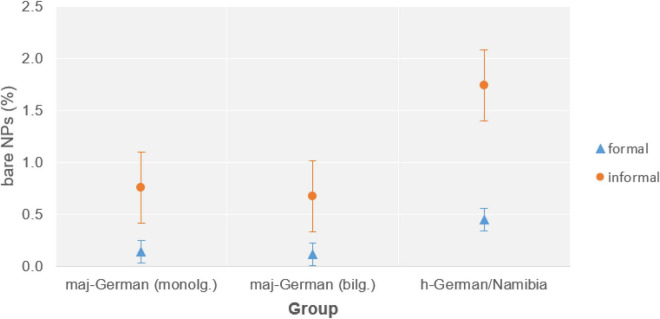
Relative frequencies of non-canonical bare NPs (percentage of all NPs) in German in informal vs. formal settings by three different speaker groups.

For non-canonical bare NPs in maj-German, we similarly found a dominance in informal data, and again this held for bilinguals as well as monolinguals. We also found this pattern in h-German in Namibia, where it was even more pronounced, cf. Figure 5,^13^ suggesting that such register differentiation can also hold in heritage grammars (but see “Register Leveling in Heritage Languages” below for h-German in the United States).

#### Register Leveling in Heritage Languages

Some patterns characteristic for informal registers in monolinguals can be generalized to formal registers in bilinguals’ heritage language use, leading to register leveling.

##### Non-canonical Bare NPs and Word Order in h-German in the United States

One example for this is h-German in the United States In the United States, speakers do not distinguish between formal and informal settings in their non-canonical word order and NP patterns, unlike (monolingual and bilingual) speakers in Germany.

For h-German V3, we find 1.89 occurrences per 100 CUs in formal settings and 1.57 in informal settings (30 and 25 occurrences, respectively). Mode, however, plays a role here, as 71% of all V3 clauses appear in the spoken mode, pointing to a more general phenomenon that we also saw in non-canonical article choice in maj-English.

H-German speakers in the United States also show a similar distribution of non-canonical bare NPs in formal and informal situations, with 0.15 and 0.17% of all NPs (13 and 10 occurrences), respectively. As the data discussed in “Noncanonical Phenomena in Informal or Spoken Registers of Majority Languages” shows, this is in contrast not only to monolingual and bilingual speakers of maj-German in Germany, but also to h-German speakers in Namibia, where non-canonical NPs were primarily associated with informal registers. This can be related to the different communicative domains for German: in Germany and Namibia, German is used in informal as well as formal settings, including formal schooling, whereas in the United States, it is mostly restricted to informal contexts.

Further evidence for register leveling comes from h-Turkish in Germany. In section “Pragmatics,” we found non-canonical placement of new referents in post-verbal position in both HSs and monolinguals. Our analysis shows that in monolingual data, all the examples of new referents in the post-verbal position occur in informal (spoken and written) settings. HSs, on the other hand, tend to place new referents in the post-verbal position across all communicative situations. Again, this can be related to communicative domains for the heritage language: similarly as for h-German in the United States, h-Turkish speakers in Germany are mostly exposed to informal language at home and with their peers and have less access to formal registers. A phenomenon typical for informal settings in mono-Turkish then spreads into formal settings in h-Turkish.

These findings, then, emphasize that non-canonical phenomena should not always be attributed to heritage languages or bilingualism in general, but they can also be a feature of informal settings in monolingual varieties. Thus, what might been seen as a consequence of language contact or attrition in a superficial account turns out to be present also in monolingual language use when different communicative situations are taken into account.

##### Non-canonical Restrictive Relative Clauses in maj- and h-Greek

A further area where we noted register leveling is the distribution of restrictive relative clauses (RCs) in Greek. RCs in Greek function as modifiers of nouns, as in other languages, and always appear in post-nominal position (Chatsiou, 2010). Greek RCs^14^ come in two types: they are either introduced by the pronoun *o opios*, literally “the who,” or by the complementizer *pu* “that.” While the pronoun is inflected for gender, case and number and agrees with the nominal head that it modifies in gender and number, *pu* bears no inflection. In the literature on Greek, we find the claim that *pu* appears mostly in colloquial speech, while the pronoun is preferred in formal registers (Holton et al., 1997: 212).

We investigated restrictive RCs for the three groups represented in our corpus: monolingual speakers of maj-Greek in Greece and bilingual speakers of h-Greek in Germany and in the United States. As Table 3 shows, *pu* RCs are more frequent than *o opios* RCs across the three speaker groups in the different countries, and this holds in both informal and formal settings.

**TABLE 3:** Quantitative data - distribution of restrictive RCs in different registers per group.

Communicative situation		h-Greek in the U.S.	h-Greek in Germany	Mono-Greek
Informal	*Pu*	165 (99.4%)	150 (82%)	183 (74.7%)
	*o opios*	1 (0.6%)	33 (18%)	62 (26.3%)
Formal	*Pu*	238 (97.9%)	190 (71.7%)	244 (64%)
	*o opios*	5 (2.1%)	75 (28.3%)	137 (36%)

Zooming in on the question of register variation, it appears that monolingual speakers actually prefer *pu* RCs even in formal settings, something that contradicts the claims in grammars of Standard Modern Greek. This points to a process of leveling even in the monolingual group. A similar pattern is exhibited by the h-groups, who also favor *pu* RCs over *o opios* RCs across settings, and a one-way ANOVA test^15^ revealed no significant differences between the three groups concerning the production of *pu* RCs in the different communicative settings.

For *o opio*s RCs, we find differences in the distribution across registers between groups, as determined by one-way ANOVA tests {formal: [*F*(*2*,*171*) = *15*.*99*, *p* < *0*.*0001*], informal: [*F*(*2*,*171*) = *8*.*877*, *p* < *0*.*0001*]}. Our data indicates that the *o opios* strategy is preferred in the formal register compared to the informal one (although at a lower rate than *pu* RCs). In this case, there is a statistically significant difference between groups, as determined by a one-way ANOVA [*F*(2, 171) = 14.50, *p* < 0.0001]. Specifically, Bonferroni-adjusted *post hoc* tests enabling pairwise comparisons revealed that the United States. group differs from the other two in the production of *o opios* RCs across the different communicative settings (*p* < 0.05). The preference for formal registers holds in particular for the monolingual group, and secondly for HSs in Germany. HSs in the United States rarely use *o opios* RCs regardless of setting [as reported also by Lithoksoou (2019), who investigated part of RUEG corpus].

Taken together, our results indicate register leveling for *pu* RCs across monolingual and bilingual speaker groups, and differences for *o opio*s RCs between different heritage speaker communities, with those in Germany aligning with monolingual speakers in Greece.

Such findings underline the relevance of considering register differentiations in bilinguals and monolinguals alike, while integrating HSs into the native speaker continuum.

## Discussion

In this manuscript, we argued for a perspective on bilingual heritage speakers as native speakers of both their languages, and demonstrated what can be gained from such an approach. We showed that recognizing heritage speakers as native speakers is justified on conceptual and theoretical grounds, and that this perspective is also a better fit for the empirical data. We presented results from a large-scale, cross-linguistic study that approached bilingual heritage speakers and monolingual speakers on equal terms, rather than using monolinguals as a yardstick for what counts as a competent “native speaker.” In line with this approach, we targeted actual language use that covered broader repertoires than just formal language, and we did so for bilingual and monolingual speakers alike. To this end, we elicited linguistic productions representative of speakers’ natural behavior in formal and informal, written and spoken communicative situations. This provided us with comparable data across registers, languages, contact-linguistic settings, and speaker groups, incorporated in an open-access corpus, the RUEG corpus.

Our findings support the integration of heritage speakers into the native-speaker continuum and show that they can shed light on language variation and change in native grammars. In a number of heritage languages, we find patterns in formal registers that do not appear in the respective majority settings for those languages. However, a closer look shows that these patterns are by no means completely absent from monolingual grammars, but can be associated with informal registers there. Patterns that might otherwise have been attributed to bilingualism could hence inform us on ongoing developments and variation in native grammars.

Our data points to a range of non-canonical patterns in monolinguals’ productions that have so far been considered absent from native grammars. We discussed non-canonical patterns of participle formation and boundary tones in Russian; verb-third and bare NPs in German; new referents in post-verbal position in Turkish; and non-canonical choices of (in-)definite articles for given vs. new referents in English. Our study showed that these patterns are not restricted to heritage speakers, but they occur systematically in monolinguals as well.

Along similar lines, we found inter-speaker variability not restricted to bilinguals either. In our data for German, monolinguals displayed a degree of lexical and morphosyntactic variation that was sometimes higher than that of bilinguals, further challenging the model of the streamlined native speaker.

In majority language use, non-canonical patterns were dominant in spoken and/or informal registers, and this was true for monolinguals and bilinguals alike. In majority-English, for example, spoken registers featured more non-canonical article choices than written registers in both monolingual and bilingual speakers, and in majority-German, non-canonical word order and bare NPs were associated with informal registers.

In several languages, though, our data points to tendencies of register leveling in heritage contexts. This is presumably due to a lack of formal schooling: heritage speakers are mostly exposed to informal language at home and often have limited access to formal registers. Hence, phenomena typical for informal settings in majority language use can spread to formal settings in heritage languages. This points to different register distributions rather than to distinct grammatical patterns in heritage speakers. Accordingly, where the heritage language is also used in formal schooling, e.g., in the case of heritage German in Namibia, patterns were similar to those found for majority language use (i.e., German in Germany). As our Greek data showed, register leveling can also occur in monolingual speakers: we found that, contrary to claims in the literature, a non-inflected relativizer is preferred in formal as well as informal registers, and we found this for monolinguals and heritage speakers alike.

Some non-canonical patterns in informal settings were more frequent in bilingual speakers. Heritage speakers can hence put a unique spotlight on internal developments. In line with this, several of the non-canonical patterns we found point to extensions of existing, salient variants and/or ongoing language change. This underscores the importance of taking into account a broader range of communicative settings, not just for heritage speakers, but also for monolinguals.

For heritage languages, our findings also indicate that the size and cohesion of speech communities can play a role. In smaller, more widely distributed communities like our populations in the United States, we sometimes found more diverging patterns. This does not imply that those options are outside native grammars, but we might see more variation. Our data point to broader options of non-canonical word order and bare NPs in heritage German in the United States, while heritage German in Namibia patterned with majority German. For heritage Russian, we found a lower frequency of participles in the United States, while heritage Russian in Germany patterns with majority Russian.

Our findings also lead us to reconsider the state-of-the art on majority languages, given recurring evidence for non-canonical patterns in monolinguals that deviate from what has been assumed in the literature so far. In the case of the intonational pattern analyzed in Russian, it was in fact the “deviant” pattern, that is, the one that differed from those described in the literature, that we found in monolinguals as well as in one of our heritage language communities, namely in Germany, but not in the other, that is, in heritage Russian in the United States.

Taken together, our findings support current calls to normalize multilingualism. Multilingualism can act as a motor of linguistic developments, and accordingly, multilingual communities can afford us a privileged view into ongoing tendencies of language variation and change. However, this does not make them an exotic, special case. Our findings put multilinguals’ language solidly within native grammars, at levels of structure as well as language use. In order to make full use of the opportunity that multilingual speakers provide for linguistic theory, we need to take into account variation in native grammars, including informal registers, in bilinguals and monolinguals alike.

## Data Availability Statement

The datasets presented in this study can be found in online repositories. The names of the repository/repositories and accession number(s) can be found below: http://doi.org/10.5281/zenodo.3236069.

## Ethics Statement

The studies involving human participants were reviewed and approved by DGfS Ethics Committee (Deutsche Gesellschaft für Sprachwissenschaft/German Society for Linguistics). Written informed consent to participate in this study was provided by participants or, in case of minors, their legal guardian/next of kin.

## Author Contributions

All authors listed have made a substantial, direct, and intellectual contribution to the work, and approved it for publication.

## Conflict of Interest

The authors declare that the research was conducted in the absence of any commercial or financial relationships that could be construed as a potential conflict of interest.

## Publisher’s Note

All claims expressed in this article are solely those of the authors and do not necessarily represent those of their affiliated organizations, or those of the publisher, the editors and the reviewers. Any product that may be evaluated in this article, or claim that may be made by its manufacturer, is not guaranteed or endorsed by the publisher.

## Footnotes

1 E.g., Rothman and Treffers-Daller (2014); Guijarro-Fuentes and Schmitz (2015), Schroeder (2016); Kupisch and Rothman (2018), Aalberse et al. (2019); Lohndal et al. (2019), Embick et al. (2020), and Flores and Rinke (2020).

2
https://www.linguistik.hu-berlin.de/en/rueg

3 Funded by Deutsche Forschungsgemeinschaft, DFG (FOR 2537). Speaker: Heike Wiese; further PIs: Artemis Alexiadou, Shanley Allen, Oliver Bunk, Natalia Gagarina, Mareike Keller, Anke Lüdeling, Judith Purkarthofer, Christoph Schroeder, Anna Shadrova, Luka Szucsich, Rosemarie Tracy, Sabine Zerbian; postdoc: Kalliopi Katsika; Ph.Ds: Katerina Iefremenko, Esther Jahns, Martin Klotz, Thomas Krause, Annika Labrenz, Maria Martynova, Katrin Neuhaus, Tatiana Pashkova, Vicky Rizou, Wintai Tsehaye, and Yulia Zuban.

4 In general before the age of 23 months, although in some cases this was extended (up to 4 years) where otherwise it would not have been possible to recruit enough speakers.

5 We define “token” as the minimal annotated unit in our corpus: a string of characters between two spaces in written text, or in the written transcription of spoken text. Thus, a token is typically a word (e.g., “dog”) or an emoji or emoticon (e.g.,:-/). Punctuation marks and filled pauses (e.g., “um”) are not included as tokens.

6 Note that the “bilingual” rows represent the same speakers on two lines – e.g., maj-German (44) and h-Greek (47) are the same speakers.

7 In cases where we were interested in the impact of multiple mixed effects and/or inter-individual variation, linear mixed models were applied. In other cases, where we simply wanted to look at frequency differences across groups, we used the non-parametric Wilcoxon rank sum test for non-normally distributed data and parametric ANOVAs for normally distributed data.

8
http://agd.ids-mannheim.de/DNAM_extern.shtml

9 See figures for maj-English in Table 1 above. Eight HSs (three with h-German, two with h-Greek, and three with h-Turkish) were excluded from the analysis due to technical reasons.

10 Even though Figure 3 contains some fine-grained prepositional subcategories (PNA and PND marking accusative/dative complements to prepositions) that go beyond the underlying dependency grammar (Foth, 2006), and dependency parses have been manually corrected, it is possible that more diversity on this level only becomes visible under consideration of more fine-grained lexicosyntactic phenomena such as constructions.

11 The TTR is transformed by the formula types/tokens × 4√tokens, or TTR × 4th root of text length (or corpus size, respectively) and serves only to adjust for text-length dependency. No claims are made to the general usefulness of approximating lexical richness through TTRs.

12 See Shadrova (2020) for a discussion of measuring coselectional constraints in corpora.

13 Note, though, that the corpus data for h-German in Namibia comes only from spoken productions (see “Morphology and Syntax” above).

14 Free RCs are excluded from this account.

15 According to the central limit theorem, the sample distribution will be approximately normally distributed as the sample size is quite large (*N* > 50 items).
