# Supplementary material for: Heritage Speakers as Part of the Native Language Continuum
Source: Front Psychol. 2022 Feb 9;12:717973. doi: 10.3389/fpsyg.2021.717973 (PMC8865415; doi:10.3389/fpsyg.2021.717973)
Supplement: Supplementary file 1 [file Data_Sheet_1.PDF]

Appendix 1: Model specifications and summaries for final boundary tones in *yes-no* questions in Russian (Section 3.1.2)

Table 1: Generalized linear mixed effects model for final boundary tone choices

Model formula:

pitch_fbt ~ country * nsyl_acc2fin + nuclear +transitivity + (1 + nsyl_acc2fin| speaker) + (1 | item), family = "binomial")

Model parameter	Estimate	SE	z	p
(Intercept)	1.9347	1.0207	1.895	.058
countryRU	0.4534	0.833	0.544	.586
countryUS	0.8527	1.215	0.702	.483
nsyl_acc2fin	-1.5591	0.776	-2.009	.045*
nuclearHL	1.772	1.5655	1.132	.258
nuclearL	2.8712	1.0892	2.636	.008**
nuclearLH	-0.2853	0.6915	-0.413	.680
transitivity	-1.2881	0.5302	-2.429	.015*
countryRU:nsyl_acc2fin	-0.52	0.8639	-0.602	.547
countryUS:nsyl_acc2fin	-3.2374	1.3039	-2.483	.013*

For the interaction of countryUS and number of syllables following the last pitch accent to the FBT, we ran a Tukey multiple comparison test.

Formula: emmeans(FBT.model, pairwise ~ country | nsyl_acc2fin)

Contrast	Estimate	SE	df	z	p
DE - RU	0.393	1	Inf	0.392	.919
DE - US	4.417	1.29	Inf	3.413	.002
RU - US	4.024	1.24	Inf	3.252	.003
