# Supplementary material for: Heritage Speakers as Part of the Native Language Continuum
Source: Front Psychol. 2022 Feb 9;12:717973. doi: 10.3389/fpsyg.2021.717973 (PMC8865415; doi:10.3389/fpsyg.2021.717973)
Supplement: Supplementary file 2 [file Data_Sheet_2.PDF]

Appendix 2: Model specifications and summaries for English "a+given" and "the+new" referents (Section 3.1.3)

Table 1: Generalized linear mixed effects model for "a+given" referents in English

Model formula:

a_given_model <- glmer (Determiner ~ Bilingualism + Setting + Mode + (1|speaker) + (1|referent), data=all_given_final, family = binomial)

The fixed effects were sum contrast coded (-0.5/0.5). The interactions of fixed effects were not included since the model with interactions returned a perfect correlation of fixed effects.

Model parameter	Estimate	SE	z	p
(Intercept)	-5.714	0.434	-13.170	< .001
Bilingualism	0.112	0.372	0.302	.763
Setting	-0.025	0.245	-0.102	.919
Mode	0.573	0.252	2.275	.023*

Table 2: Generalized linear mixed effects model for "the+new" referents in English

Model formula:

the_new_model = glmer (Determiner ~ Bilingualism * Setting * Mode + (1|speaker) + (1|referent), data=all_new_final, family = binomial, control = glmerControl(calc.derivs=FALSE))

The fixed effects were sum contrast coded (-0.5/0.5).

Model parameter	Estimate	SE	z	p
(Intercept)	-2.886	0.424	-6.808	< .001
Bilingualism	0.029	0.236	0.124	.902
Setting	-0.148	0.140	-1.059	.290
Mode	0.338	0.139	2.431	.015*
Bilingualism:Setting	0.311	0.278	1.121	.262
Bilingualism:Mode	-0.189	0.278	-0.682	.495
Setting:Mode	-0.138	0.277	-0.499	.618
Bilingualism:Setting:Mode	0.025	0.553	0.045	.964
