# Supplementary material for: Heritage Speakers as Part of the Native Language Continuum
Source: Front Psychol. 2022 Feb 9;12:717973. doi: 10.3389/fpsyg.2021.717973 (PMC8865415; doi:10.3389/fpsyg.2021.717973)
Supplement: Supplementary file 3 [file Data_Sheet_3.PDF]

Appendix 3: Abbreviations used for investigation of variation across speaker groups of German (Section 3.2)

Schema modified from Foth (2006); modified labels are marked with an asterisk (*).

ADV	–	adverb
*DIRP	–	prepositional phrase, directional
*DIRPP	–	prepositional phrase, directional, preposition specified by the verb
EXPL	–	expletive *es* (‚it‘)
NEB	–	subordinate clause
OBJA	–	accusative object
OBJD	–	dative object
OBJP	–	prepositional object (preposition specified by the verb)
PN	–	nominal complement to a preposition without overt case marking
*PNA	–	nominal complement to a preposition, accusative
*PND	–	nominal complement to a preposition, dative
*PNG	–	nominal complement to a preposition, genitive
PP	–	prepositional phrase
PRED	–	predicate
REL	–	relative clause
SUBJ	–	subject
